# Acquired von Willebrand syndrome in cardiogenic shock patients on mechanical circulatory microaxial pump support

**DOI:** 10.1371/journal.pone.0183193

**Published:** 2017-08-14

**Authors:** Ulrike Flierl, Jörn Tongers, Dominik Berliner, Jan-Thorben Sieweke, Florian Zauner, Christoph Wingert, Christian Riehle, Johann Bauersachs, Andreas Schäfer

**Affiliations:** Hannover Medical School, Department of Cardiology and Angiology, Cardiac Arrest Center and Advanced Heart Failure Unit, Hannover, Germany; GERMANY

## Abstract

Early use of mechanical circulatory support, e.g. veno-arterial extracorporeal membrane oxygenation (ECMO) or left ventricular unloading by microaxial pump in refractory cardiogenic shock is recommended in current guidelines. Development of acquired von Willebrand Syndrome (AVWS) in patients with left ventricular assist devices (LVADs) and ECMO has been reported. There is an increasing number of patients treated with the Impella^®^ CP microaxial pump for left ventricular unloading. However, the prevalence of AVWS in these high risk patients is unknown and needs to be determined. We therefore screened 21 patients (68 ± 11years) treated with Impella^®^ (17 for cardiogenic shock, 4 for protected PCI) for the presence of AVWS by determining von Willebrand factor multimers, VWF collagen binding capacity and VWF antigen. During the time course of Impella^®^ support, 20/21 patients (95%) developed AVWS (mean duration of support: 135 ± 114 hours, mean time from device implantation to first diagnosis of AVWS: 10.6 ± 10.8 hours). Our data indicate that AVWS is a common phenomenon during left ventricular unloading via microaxial pump support. Thus, AVWS has to be considered as contributing factor for potential bleeding complications in this high risk patient population, especially in the context of dual antiplatelet therapy.

## Introduction

Mortality of cardiogenic shock (CS) patients remains high despite improvement of intensive care strategies. Hence, current guidelines recommend the early use of mechanical circulatory support (MCS) in refractory CS [1].

Amongst others, options of MCS comprise veno-arterial extracorporeal membrane oxygenation (va-ECMO) bypassing the right and left ventricle [2] and microaxial pump for left ventricular unloading [3] such as the Impella^®^ family. Microaxial pumps can easily be implanted under fluoroscopic guidance directly in the cath lab via transfemoral access. Blood is drawn from the left ventricle cavity and expelled above the aortic valve into the ascending aorta. Different Impella^®^ devices are available varying in size and maximum flow capabilities [4]. As coronary ischemia is a major cause of CS, rapid revascularization is the treatment of choice. Consequently, dual antiplatelet therapy as well as systemic anticoagulation to prevent clotting of the device is indispensable on mechanical support. As triple therapy predisposes to bleeding complications, distinct therapy options depending on the underlying mechanism would be desirable.

Acquired von Willebrand syndrome (AVWS) is a bleeding disorder caused by structural or functional alterations of von Willebrand factor (VWF) commonly attributable to an underlying disease, such as hematological and autoimmune disorders and cardiovascular diseases. The latter group, for example, comprises patients with aortic valve stenosis [5] and the increasing cohort of permanent left ventricular assist device (LVAD) patients [6, 7]. In LVAD patients, shear stress-induced conformational change and subsequent proteolytic cleavage [8] is the proposed mechanism for AVWS. As Impella^®^ microaxial pumps achieve very high shear rates we investigated the prevalence of AVWS in this patient cohort.

## Methods

### Patients

In this observational study, 21 consecutive patients with microaxial pump support Impella CP^®^ were investigated between August 2016 and March 2017. Blood samples (2 x 3 mL) were drawn using commercial tubes containing 3.2% citrate which were directly sent to a certified laboratory where standardized testing for AVWS was performed. The collection of blood samples for research had been approved by the ethics committee at the Hannover Medical School.

### Laboratory testing

AVWS analysis was performed in a core facility (Medilys Laboratory, Asklepios Klinik Altona, Hamburg, Germany) analyzing VWF antigen (VWF:Ag), VWF collagen binding activity (VWF:CB) and plasma VWF by electrophoresis of VWF to determine its multimeric structure. The VWF multimer pattern was analyzed using low-resolution agarose gel electrophoresis (1.2% lgt-agarose) [9]. Moreover, VWF activity (VWF:Act) assay was performed measuring agglutination of polystyrol particles coated with GPIb and addition of recombinant GPIb. The ratios of VWF:CB/VWF:Ag and VWF:Act/VWFAg were performed to quantify the functional capability of VWF. A ratio > 0.8 represents physiologic functionality of VWF [9]. In all patients VWF diagnostics was performed during Impella^®^ treatment, in 12 patients, samples were available before as well as after Impella^®^ implementation.

### Anticoagulation and / or antiplatelet therapy

All patients were treated with unfractionated heparin during microaxial pump support aiming at an activated clotting time [ACT] of 160–180 sec. In addition to effective anticoagulation, 17 of the patients were on dual antiplatelet therapy after drug-eluting stent implantation.

### Bleeding events

Bleeding events were assessed by the TIMI bleeding classification as previously reported [10].

### Statistical analysis

Values are given as mean ± standard deviation (SD). For comparison between different time points Friedman’s nonparametric test for matched data was used followed by Dunn’s multiple comparisons test. Data were analyzed using GraphPad Prism 6.0 (GraphPad Software, Inc., La Jolla, CA).

## Results & discussion

Patient characteristics are shown in Table 1. Mean age was 68 ± 11 years, and mean duration of Impella^®^ support was 135 ± 114 hours. In addition to therapeutic administration of unfractionated heparin (target ACT 160–180 sec), 17 patients were on dual antiplatelet therapy (n = 6 aspirin + clopidogrel, n = 11 aspirin + prasugrel) due to prior coronary stent implantation (Table 1).

**Table 1:** Patient characteristics.

N	21
Age (years)	68 ± 11
Sex (male)	17 (81%)
Indication for microaxial pump support	
Cardiogenic shock	17
• Cardiopulmonary Resuscitation	9/17 (53%)
- OHCA	7/17
- IHCA	2/17
• Protected PCI	4
Duration of Impella^®^ support (hrs)	135 ± 114
Implementation of hypothermia (32°C for 24hrs)	8/21 (30%)
Antiplatelet therapy	19
• aspirin–prasugrel	11
• aspirin–clopidogrel	6
• aspirin only	2
Bleeding events	
• TIMI bleeding	
- None	3/21 (14.3%)
- Minimal	5/21 (23.8%)
- Minor	13/21 (61.9%)
- Major	0/21
Transfusion of blood products	
• Red blood concentrates	5 ± 6 (13/21)
• Platelet concentrates	0.4 ± 0.8 (3/21)
Intrahospital survival	14/21 (66.7%)

Values as number (%) of observation, means ± standard deviation

OHCA = out-of-hospital cardiac arrest

IHCA = in-hospital cardiac arrest; PCI = percutaneous coronary intervention

**Fig 1:**
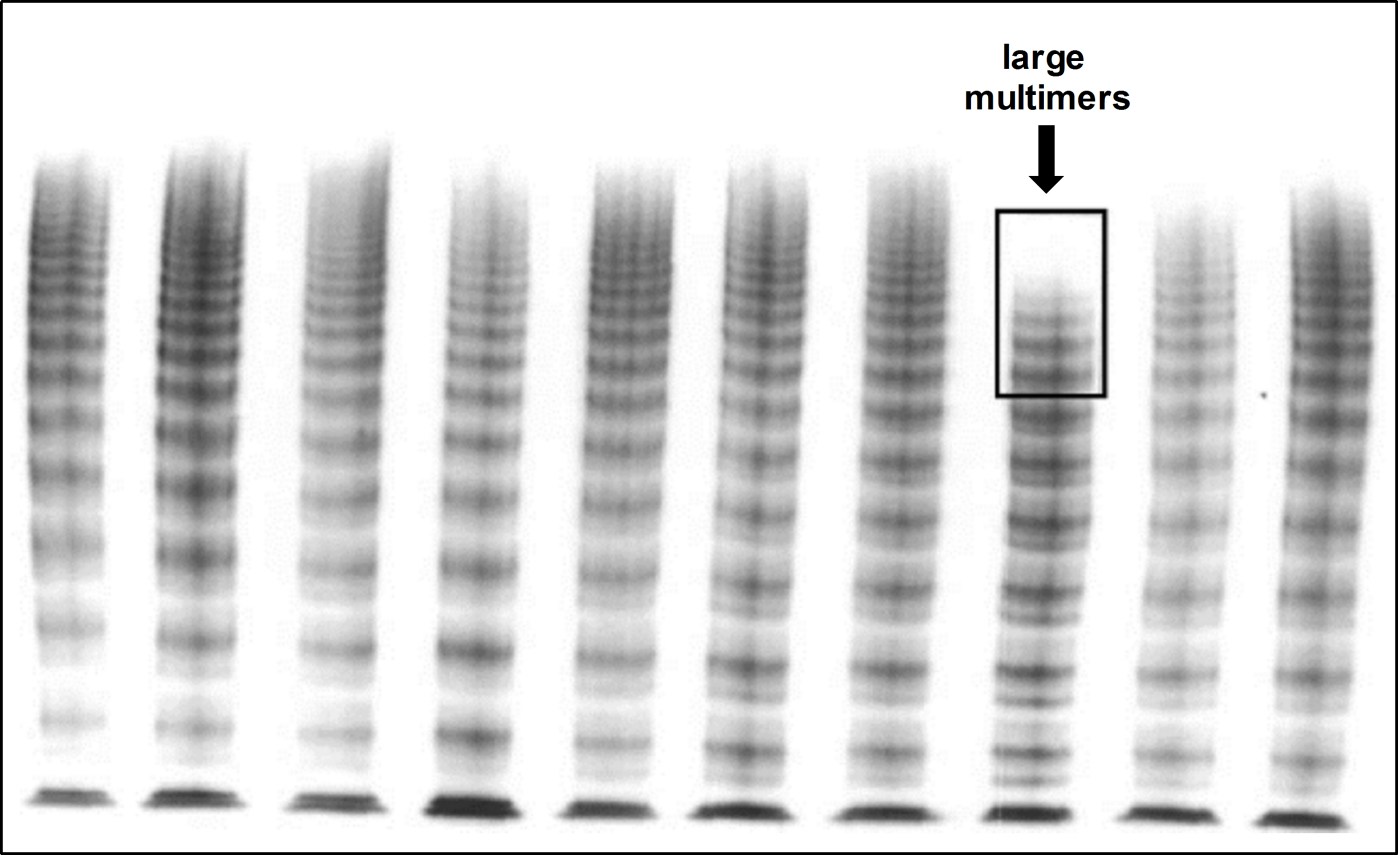
Representative electrophoresis. Low-resolution gel (1.2%) of plasma from a patient with Impella^®^ support (marked lane) compared to other plasma samples. Very large VWF multimers are missing, large multimers are reduced.

**Fig 2:**
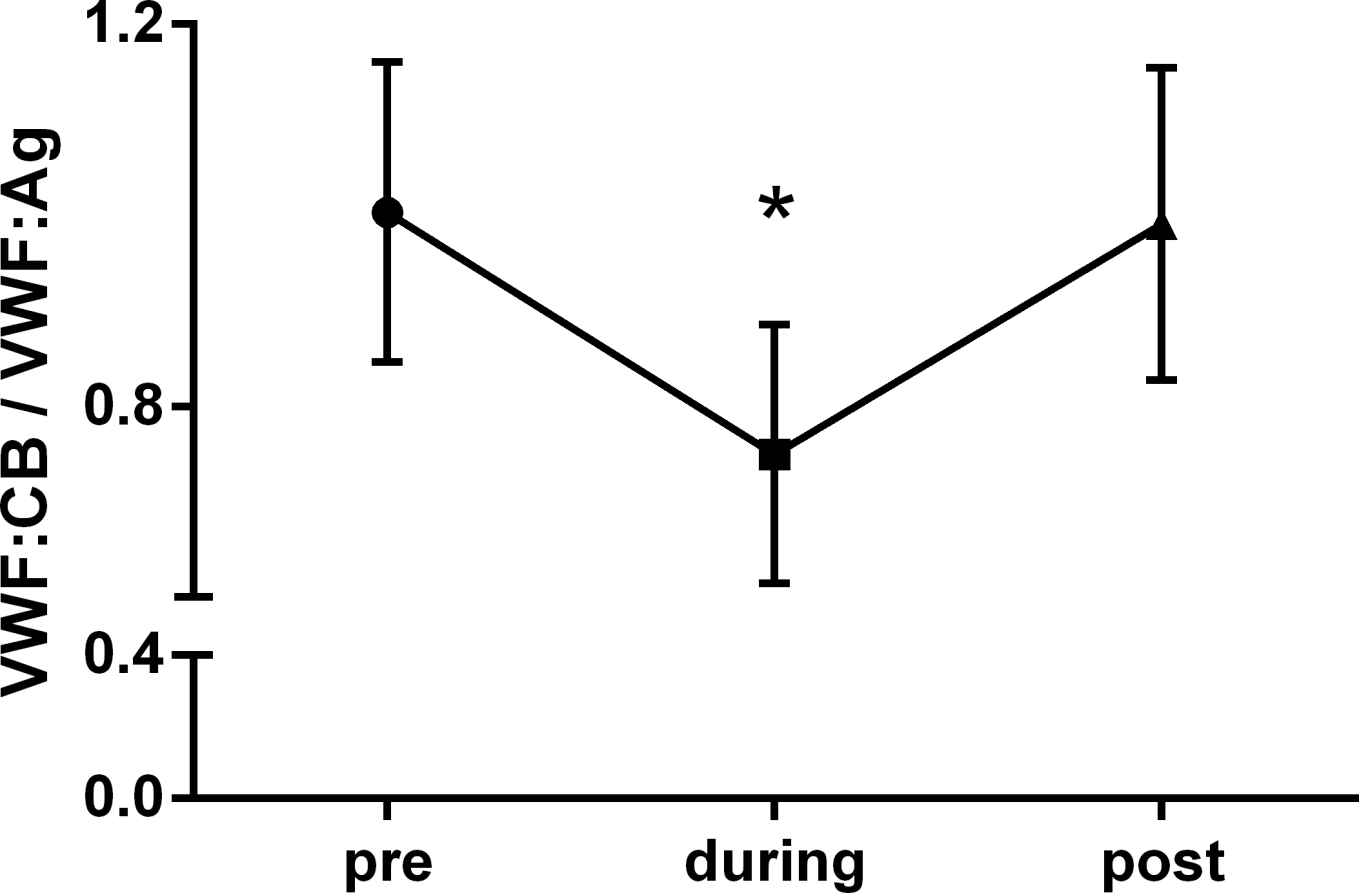
Course of VWF:CB / VWF:Ag ratio. Significant reduction of VWF:CB / VWF:Ag ratio during Impella^®^ support—and normalization after cessation of left ventricular unloading. n = 12, *p<0.05 vs pre / post Impella^®^ therapy.

**Fig 3:**
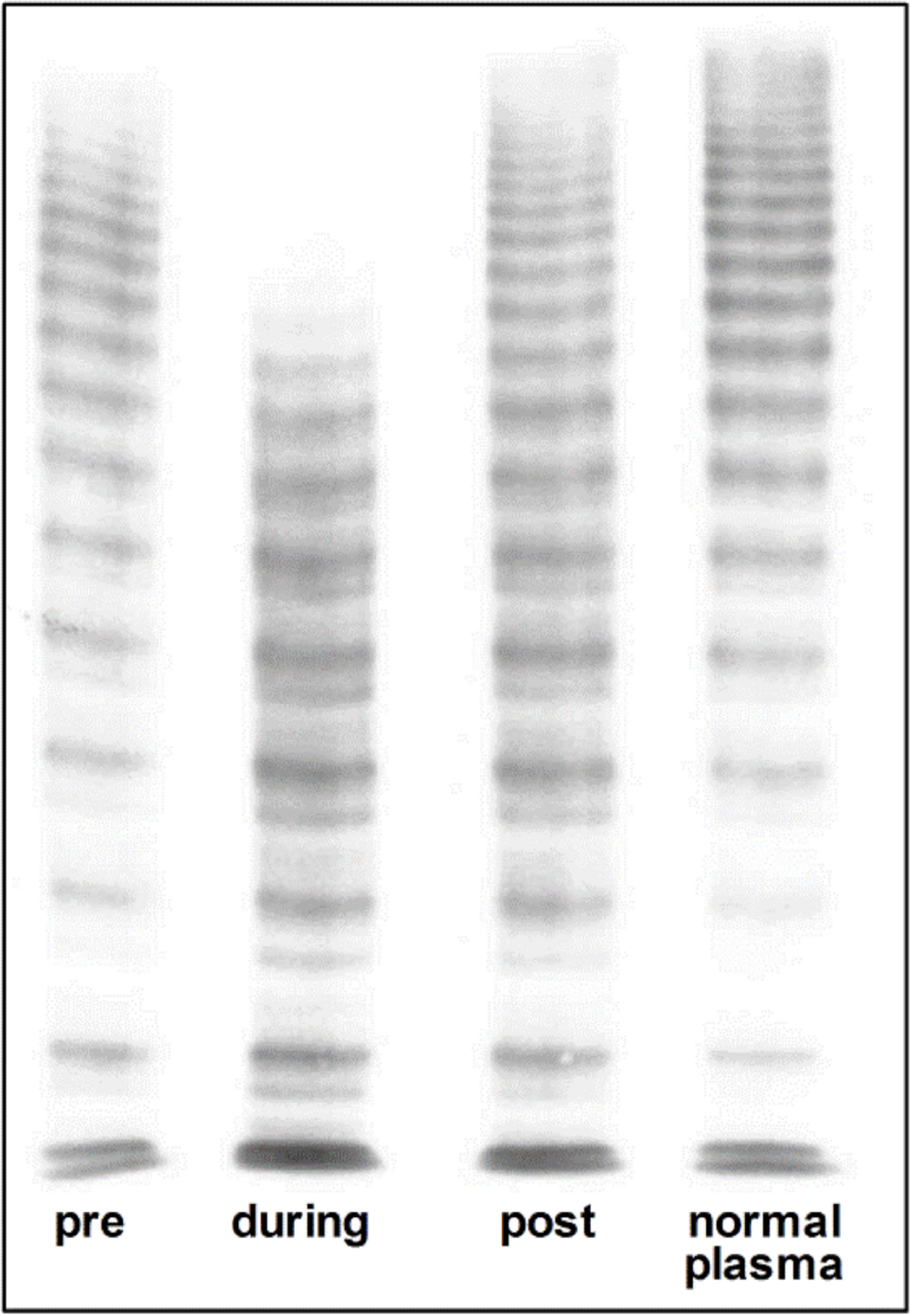
Characteristic electrophoresis at different time points. Characteristic low-resolution gel (1.2%) of a patient with Impella^®^: Very large multimers are absent and large multimes are reduced during microaxial pump support. Existence / recovery of large multimers is documented before and after mechanical left ventricular unloading. The right lane illustrates a characteristic trace of a plasma sample from a healthy person.

Ninety-five percent of patients were diagnosed with VWS, i.e. loss of very large multimers (Fig 1) and concomitant significant decrease of VWF:CB–VWF:Ag ratio (Fig 2). Occurrence of VWS was rapid, mean time from device implantation to first assessment of von Willebrand parameters was 10.6 ± 10.8 hours. In one patient, AVWS was detected as soon as 93 min post beginning of left ventricular unloading. For 12 patients, VW diagnostics were performed before as well as after removal of Impella^®^ (mean time 25 hours post implantation and 58 hours post explantation). In all these patients, there was a significant increase in VWF:CB / VWF:Ag ratio compared to the ratio during microaxial pump support (Fig 2, Table 2), which is also reflected in regression of AVWS, i.e. recovery of large and very large multimers by electrophoresis (Fig 3). No correlation between level of left ventricular unloading and amount of high molecular weight multimers could be observed (data not shown). This is in line with a previous report of LVAD patients where device speed did not significantly influence the extent of VWF degradation [11]. Bleeding complications and the need for transfusion were more frequently observed in CS patients requiring device therapy over a longer period of time. No major bleeding events according to the TIMI classification could be observed. However, minor bleedings were frequent (Table 1), most often located in the nasopharyngeal area.

**Table 2:** Diagnostics of von Willebrand syndrome.

Presence of AVWS	20 / 21 (95%)
Time device implant–first diagnosis of AVWS (hrs)	10.6 ± 10.8
Platelet count (10^3^ / μL)	160 ± 61
VWF: CB (%)	213 ± 82
VWF: Ag (%)	265 ± 106
VWF: Act (%)	183 ± 56
VWF: CB / VWF: Ag Ratio	0.82 ± 0.1
VWF: Act / VWF: Ag Ratio	0.71 ± 0.2

VWF = von Willebrand factor; CB = collagen binding activity; Ag = antigen; AVWS = acquired von Willebrand Syndrome

The presence of AVWS is a well-described observation in LVAD patients [12] and patients on ECMO [13, 14]. Recent data suggest two mechanisms to be involved in this phenomenon: shear stress induced mechanical damage and, more importantly, enzymatic cleavage by ADAMTS-13 (a disintegrin and metalloproteinase with a thrombospondin type 1 motif, member 13) [15]. Interestingly, inhibition of ADAMTS-13 significantly decreased VWF degradation during supraphysiological shear stress without affecting platelet activity in an *ex vivo* setting [16].

Here, we for the first time describe the presence of AVWS in patients treated with a microaxial pump for left ventricular unloading. 17 of the 21 investigated patients were treated for cardiogenic shock, 7 of them after previous out-of-hospital resuscitation, 2 after prior in-hospital resuscitation. All of these patients developed AVWS and had minor bleeding complications according to the TIMI classification [10] (Table 1). As resuscitated patients were routinely subjected to therapeutic hypothermia (32°C core temperature) following cardiac arrest, and hypothermia by itself might increase the likelihood of bleeding, we cannot definitively relate the observed AVWS to the bleeding phenotype in this group of patients. However, apart from the patients undergoing protected PCI with only short-time microaxial pump support, almost all cardiogenic shock patients required red blood cell concentrates independently of hypothermia implementation. Triple anticoagulation by itself undoubtedly increases the risk of bleeding and cannot be withheld in patients with recent coronary intervention and microaxial pump support as presented in this cohort. It is unclear to what extent the presence of AVWS contributes to observed bleeding complications. Still, it is important to raise the awareness that genesis of bleeding is multifactorial.

In summary, it is tempting to speculate that AVWS alone might not be relevant in stable patients. However, in critically ill and hemodynamically unstable patients the development of AVWS should be considered as predisposing factor. This cohort most often requires dual antiplatelet therapy due to acute coronary intervention and frequently experiences a significant decrease in platelet count for various reasons [17]. As AVWS might be one factor contributing to bleeding complications, a specific treatment option, i.e. ADAMTS-13 inhibition, which might become available in the near future, would be desirable.

## Supporting information

S1 File Raw data of von Willebrand syndrome diagnostics. (PZFX)

S2 File Raw data of patient characteristics. (XLSX)
